# Lipopolysaccharide induces SBD-1 expression via the P38 MAPK signaling pathway in ovine oviduct epithelial cells

**DOI:** 10.1186/s12944-016-0294-4

**Published:** 2016-08-11

**Authors:** Qi Li, Fuxiang Bao, Dafu Zhi, Moning Liu, Qin Yan, Xinxin Zheng, Lixin Ren, Shan Cong, Yan Li, Guifang Cao

**Affiliations:** 1 College of Veterinary Medicine, Inner Mongolia Agricultural University, No. 306, Zhaowuda Road, Huhhot, 010018 People’s Republic of China; 2 Key Laboratory of Clinical Diagnosis and Treatment Techniques for Animal Disease, Ministry of Agriculture, No. 306, Zhaowuda Road, Huhhot, 010018 People’s Republic of China

**Keywords:** Ovine oviduct epithelial cells, SBD-1, LPS, P38 MAPK, TLR4

## Abstract

**Background:**

Beta defensins are secreted from ovine oviduct epithelial cells (OOECs) in response to microbial infection, and are potential alternatives to antibiotic agents in the treatment of microorganism infection, particularly given the abuse of antibiotic agents and the increasing number of drug-resistant bacteria. The aberrant expression of defensins may result in disorders involving organ and oviduct inflammation, such as salpingitis.

**Methods:**

In the present study, we investigated the effects of LPS on the mRNA expression levels of sheep β-defensin-1 (SBD-1) in ovine oviduct epithelial cells. The OOECs in vitro culturing system were established and treated with different concentrations of LPS for indicated time. In addition, MAPK inhibitors and TLR4 antibodies were pretreated to investigate the potential mechanism which involves in LPS regulating SBD-1 expression.

**Results:**

LPS markedly upregulated SBD-1 expression in a concentration- and time-dependent manner. Treatment with 100 ng/mL LPS resulted in the phosphorylation of JNK, ERK and P38 MAPK. Interestingly, the LPS stimulated SBD-1 expression was attenuated by pretreatment with the P38 MAPK inhibitors SB203580 and SB202190 but not the JNK inhibitor SP600125, while the ERK inhibitor PD98059 had a minor effect. Furthermore, treatment with a Toll-like receptor 4 (TLR4) neutralizing antibody significantly decreased P38 MAPK phosphorylation and LPS induced SBD-1 expression.

**Conclusions:**

Together, these findings suggest that SBD-1 is upregulated by LPS via the TLR4 receptor, mainly through the P38 MAPK signaling pathway in ovine oviduct epithelial cells to protect the ovine oviduct epithelium from pathogen invasion.

**Electronic supplementary material:**

The online version of this article (doi:10.1186/s12944-016-0294-4) contains supplementary material, which is available to authorized users.

## Background

In recent years, great attention has been paid to defensins, which are a group of broad-spectrum antimicrobial agents and secreted from epithelial cells in response to microbial infection and stimulation from the main component of microbial pathogens. The aberrant expression of defensins may result in disorders involving organ and oviduct inflammation, such as salpingitis [1]. Defensins are potential alternatives to antibiotic agents in the treatment of microorganism infection, particularly given the abuse of antibiotic agents and the increasing number of drug-resistant bacteria, as well as very high infection and mortality rates in the sheep industry.

Defensins are a set of small-molecular-weight peptides that usually consist of 29–42 amino acids and play an important role in both the innate immune system and the adaptive immune system in mammals [2–4]. As an important component of the innate immune system, defensins have a lethal effect on both gram-negative and gram-positive bacteria, as well as on viruses and fungi [5–7]. The bactericidal effect of defensins is closely related to their specific amphiphilic structure and positive charge. Positively charged defensins can bind to the negatively charged bacterial surface. The hydrophobic ends of defensins can bind to the phospholipid membrane of bacteria, damage its structure and cause the efflux of bacterial contents, which leads to bacterial death [8, 9].

Defensins have been detected in many species, including bovine, ovine, porcine, and human [10]. Based on different structural characteristics, defensins are divided into three main classes: α-, β-, and θ-defensins. So far, only beta defensin-1 (BD-1) and beta defensin-2 (BD-2) have been identified in sheep and are mapped to chromosome 26, which consists of two exons and one intron [11, 12]. The two exons separately encode the signal sequence and the mature peptide [13]. In our previous study, it was found that SBD-1 was widely expressed in ovine oviduct epithelial cells instead of the connective tissue, lamina propria or muscular layer of the sheep reproductive tract. Also, recombinant expressed SBD-1 could significantly inhibit *S. flexneri, E. coli, P. vulgaris, S. aureus* and *P. aeruginosa* in vitro [14, 15].

Although defensins have a significant anti-microorganism effect on innate immunity, the mechanisms which regulate the expression of defensins in ovine oviduct epithelial cells remain poorly understood. Playing a pivotal role in regulating defensin expression, mitogen-activated protein kinases (MAPKs) may be involved in the defensin-induced anti-microorganism effects. MAPKs are a family of serine/threonine protein kinases which includes three main members: extracellular signal-regulated kinase (ERK), c-Jun N-terminal kinase (JNK), and P38 MAPK. MAPKs play a significant role in a variety of physiological processes, such as cell proliferation, differentiation and apoptosis. Furthermore, studies with several cell culture systems indicate that lipopolysaccharide (LPS) can activate P38 MAPK, ERK, and JNK signaling [16].

LPS, a major integral component of the outer membrane of gram-negative bacteria, is considered one of the most potent initiators of inflammatory cytokines [17]. Toll-like receptor 4 (TLR4) was critical in the LPS-stimulated immune reaction. Mammalian TLR4 adapted primarily to subserve the recognition of LPS and presumably transfer the LPS signal across the plasma membrane [18, 19]. TLR4 knock-out mice were unresponsive to LPS [20].

The activation of TLR4 results in the activation of multiple signaling pathways, including mitogen-activated protein kinases (MAPKs), which lead to the induction of antimicrobial responses [21]. An injury-induced increase in TLR4 reactivity was mediated by the enhanced activation of the P38 signaling pathway [22]. The protective effect of estradiol on Kupffer cell function was mediated by the downregulation of TLR4-dependent p38 MAPK and NF-kB signaling following trauma-hemorrhage which prevented the systemic release of cytokines [23].

This study established an ovine oviduct epithelial cells in vitro culturing system and treated the cells with LPS, with or without MAPK inhibitors and an anti-TLR4 antibody. Quantitative RT-PCR, western blotting and immunohistochemistry were performed to observe the induction of SBD-1 expression by LPS in ovine oviduct epithelial cells, in order to investigate the involvement of the MAPK signaling pathway and to determine the cellular localization of P38 MAPK. This study lays a solid foundation to the understanding on the pathogenesis of oviduct inflammation, such as salpingitis, and the reaction of the host immune system to microbial invasion.

## Methods

### Reagents

LPS (Cat. No. L2880), the P38 MAPK inhibitors SB203580 (Cat. No. S8307) and SB202190 (Cat. No. S7067), the JNK inhibitor SP600125 (Cat. No. S5567), and the ERK1/2 inhibitor PD98059 (Cat. No. P215) were purchased from SIGMA-ALDRICH. The anti-P38 antibody (Cat. No. SC-7149), the anti-P-P38 antibody (Cat. No. SC-101759), the anti-P-JNK antibody (Cat. No. SC-6254), HRP-conjugated anti-rabbit secondary antibody (Cat. No. SC-2004), and HRP-conjugated anti-mouse secondary antibody (Cat. No. SC-2005) were obtained from Santa Cruz. The anti-ERK antibody (Cat. No. SC-94) and the anti-P-ERK antibody (Cat. No. SC-7383) were obtained from Zhongshan Golden Bridge. The anti-JNK antibody (Cat. No. 3708) was purchased from Cell Signaling Technologies. The anti-Cytokeratin 18 antibody (Cat. No. MAB-0182) was obtained from Fuzhou Maixin Biotech. The anti-TLR4 antibody (Cat. No. 16-9917-82) was obtained from eBioscience. All of the other chemicals that were used were of analytical grade and obtained from commercial sources.

### Animals

All of the sheep used in this study were 13–15 months old and purchased from Tecon Group in Urumqi (Xinjiang Autonomous Region, PR China). The sheep had free access to food and water. All of the experimental procedures were performed in accordance with the institutional and national guidelines and regulations and approved by the Animal Care and Use Committee of Inner Mongolia Agriculture University. Euthanasia was performed by the intravenous injection of a barbiturate overdose, and followed by exsanguination and the immediate removal of the oviducts.

### Cell culture

Ovine oviduct epithelial cells were isolated according to a widely used approach under sterile conditions. In brief, the collected ovine oviducts were immediately placed into cold PBS that was supplemented with penicillin and streptomycin. After being bisected longitudinally, the oviducts were immersed into Hank’s Balanced Salt Solution (HBSS) (Cat. No. H6648, Sigma, USA) with 0.05 % pancreatin (Cat. No. P3292, Sigma, USA) and 0.02 % EDTA (Cat. No. E6758, Sigma, USA) for 12 min at 37 °C. The inner surface of the oviducts was lightly scraped using a scalpel in HBSS. The scraped material was agitated and centrifuged at 800 × g for 3 min and resuspended in PBS. This step was repeated several times until the HBSS became clear. The cells were transferred into 75-cm^2^ cell culture flasks with phenol red-free DMEM/F12 (Cat. No. 11320033, Gibco, USA) that was supplemented with 20 % heat-inactivated FBS (Cat. No. F9665, Sigma, USA) in addition to 100 μg/mL streptomycin and 100 U/mL penicillin (Cat. No. 10378016, Gibco, USA). Once grown to 80 % confluence, the cells were transferred into six-well plates and maintained in a humidified atmosphere of 5 % CO_2_ and 95 % air.

### Immunofluorescence

Ovine oviduct epithelial cells were isolated and cultured in DMEM/F12 with 20 % heat-inactivated FBS for 24 h, and washed five times with PBS, and fixed with 4 % paraformaldehyde for 20 min at RT. After being fixed, the cells were washed with ice-cold PBS twice and blocked with 10 % normal goat serum (Cat. No. sc-2043, Santa Cruz, USA) plus 1 % bovine serum albumin (Cat. No. V900933, Sigma, USA) in PBS for 1 h at RT. Thereafter, the cells were incubated with anti-CK18 antibody (Cat. No. MAB-0182, Fu Zhou Mai Xin, China) overnight at 4 °C. After washed three times with PBS, The slides were incubated with Alexa Fluor 488 conjugated secondary antibody (Cat. No. ab150113, Abcam, diluted 1:100, Abcam, USA) at 37 °C for 1 h. The cell nuclei were stained with Hoechst 33342 (diluted 1:100, Cat. No. C1026, Beyotime, China) for 10 min at RT. Fluorescent images were obtained using an inverted fluorescence microscope (Olympus, Japan).

### Western blotting

The total protein of the ovine oviduct epithelial cells was extracted with M-PER™ Mammalian Protein Extraction Reagent (Cat. No. 78501, Thermo, USA) according to the manufacturer’s instructions. The protein concentrations were measured using a BCA assay kit (Cat. No. 23250, Thermo, USA). The proteins were separated with 10 % SDS-PAGE and then transferred polyvinylidene fluoride membranes (Cat. No. ISEQ00010, Millipore, USA). After the transfer, the membranes were incubated for 1 h at RT in 5 % bovine serum albumin (BSA) in Tris-buffered saline with 0.1 % Tween-20 and then incubated overnight at 4 °C with the primary antibodies against P38 (1:500), P-P38 (1:200), JNK (1:500), P-JNK (1:200), ERK (1:200) and P-ERK (1:200). After washing in TBST, the membranes were incubated with the appropriate secondary antibody in TBST. Finally, the membranes were visualized using a Signal Chemiluminescent Detection System (GE Healthcare, USA). The optical density of each band was analyzed by using the ImageJ (version 1.44) software.

### RNA extraction and real-time qPCR

The total RNA of ovine oviduct epithelial cells was extracted with the TRIzol Reagent (Cat. No. 9109, TaKaRa, Japan) according to the manufacturer’s instructions. Reverse transcription was performed using the Prime-Script™ RT Reagent Kit (Cat. No. RR047A, TaKaRa, Japan). Real-time PCR was performed using the SYBR® Premix Ex Taq™ II Kit (Cat. No. RR820A, TaKaRa, Japan) on a CFX96 Real-Time PCR System (Bio-Rad, USA). The relative quantity of each gene was analyzed by the 2^-△△Ct^ method as previously reported [24] and normalized to the endogenous expression of β-actin. Gene specific primer sequences for qRT-PCR were shown in Table 1.

**Table 1:** The primers that were used in the study

Gene	Gene ID	Primer (5-3′)	Product size
SBD-1	2231304		
F		GGCTCCATCACCTGCTCCTC	206 bp
R		CGTCTTCGCCTTCTGTTACTTCTT	
SBD-2	100505451		
F		CTGCTCCTCGTGCTCTTCTT	98 bp
R		CAGATGCCTTTCTTCCAACG	
β-actin	443052		
F		GTCACCAACTGGGACGACA	208 bp
R		AGGCGTACAGGGACAGC	
TLR4	554263		
F		AGAAACCTCCGCTACCTTGA	130 bp
R		CAGGGAGCAAGTTGTTCTGA	

### Immunohistochemistry

Ovine oviducts were fixed in 4 % paraformaldehyde for 48 h, embedded in paraffin and sectioned to a thickness of 5 μm. After de-waxing, rehydration, and high-temperature antigen retrieval with 0.01 % sodium citrate buffer (pH 6.0), the sections were blocked with 10 % normal goat serum and immunostained with anti-P38 (1:100), anti-P-P38 (1:100) or non-immunized rabbit serum or mouse serum overnight at 4 °C. Subsequently, the sections were incubated with biotinylated secondary antibody and avidin-biotin-peroxidase (SP-9000, Zhong Shan Golden Bridge, China) before being exposed to diaminobenzidine (ZLI-9033, Zhong Shan Golden Bridge, China) for 1 min and counterstained with haematoxylin (ZLI-9643, Zhong Shan Golden Bridge, China). Non-immunized rabbit serum or mouse serum were used as controls.

### Statistical analysis

All of the experiments were repeated at least three times. The data are presented as the mean ± SD and were analyzed by a *t*-test. The *p* values < 0.05 were considered significant. *p < 0.05, **p < 0.01 (*t*-test) vs. control group. †*p* < 0.05, ††*p* < 0.01 (*t*-test) vs. LPS group.

## Results

### LPS induces SBD-1 mRNA expression

**Fig. 1:**
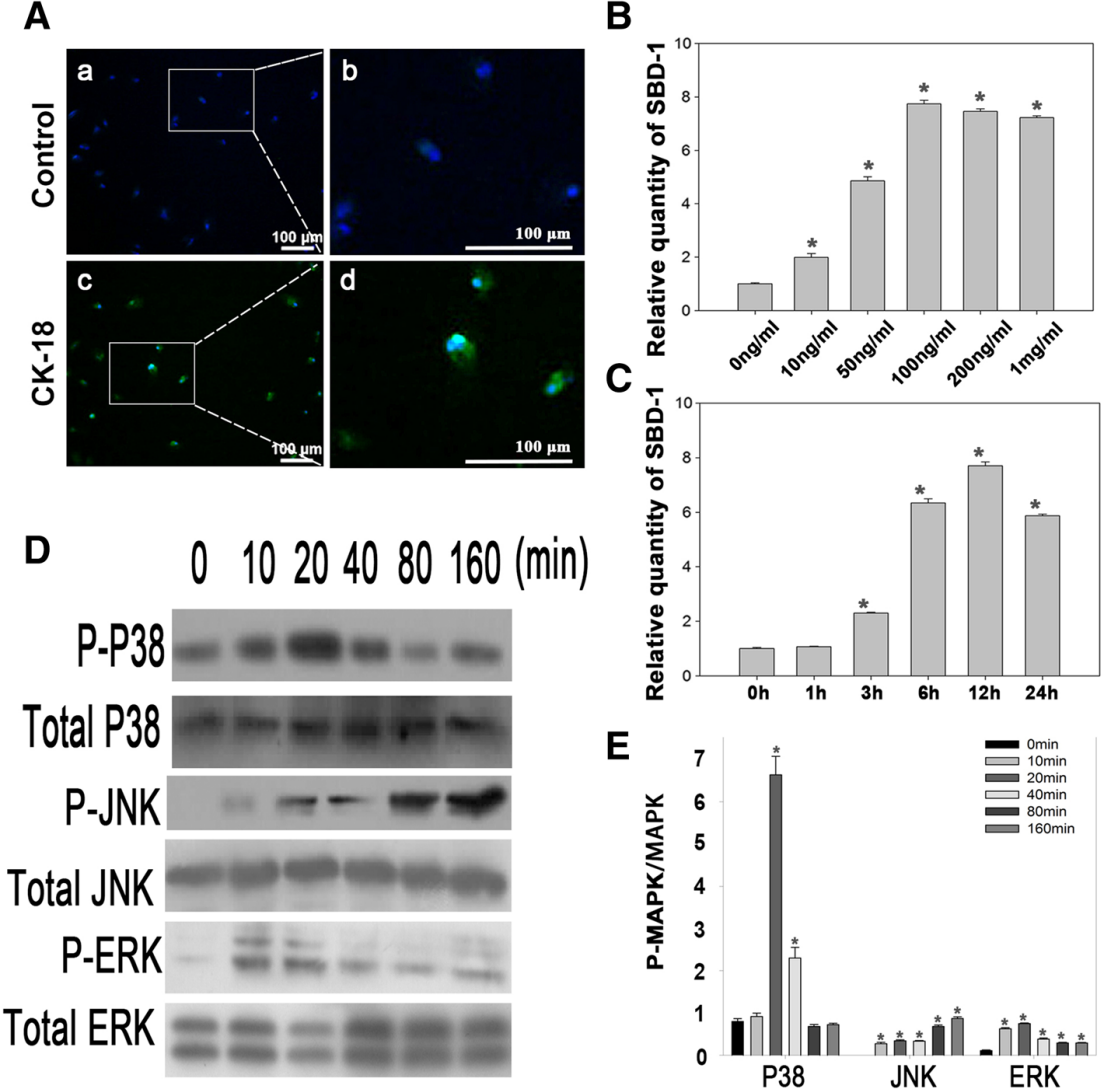
LPS induces SBD-1 mRNA expression and activates the MAPK signaling pathway in ovine oviduct epithelial cells. **a** Immunofluorescence was performed to identify ovine oviduct epithelial cells. After 24 h in culture, cells were stained for an ovine oviduct epithelial cell marker CK-18 (*green*). Hoechst 33342 (*blue*) was used to counterstain the nuclear DNA. An isotype-matched IgG was used as the negative control. Scale bar: 100 μm. **b** Ovine oviduct epithelial cells (OOECs) were treated with the indicated concentrations of LPS for 24 h and compared with untreated controls. The QRT-PCR analysis showed that LPS induces SBD-1 expression in a concentration-dependent manner. **c** OOECs were treated with LPS (100 ng/mL) for various time intervals and compared with untreated controls. The QRT-PCR results indicated that LPS induces SBD-1 expression in a time-dependent manner. **d** OOECs were treated with LPS (100 ng/mL) and harvested at the indicated time points. Whole-cell lysates were prepared and used for western blot analysis with MAPK and P-MAPK antibodies. **e** A densitometric analysis of the optical density (OD) of different p-MAPKs relative to the OD of MAPKs. All of the experiments were repeated at least three times. *p < 0.05 vs control (*t*-test)

To identify whether the isolated cells were oviduct epithelial cells, sections of cells were immunolabeled with oviduct epithelial cell marker, CK-18 (green), and nuclear marker, Hochest 33342 (blue) by immunofluorescence analysis. After 24 h in culture, almost 95 % cells exhibited strong green fluorescence with a fusiform pattern (Fig. 1a), which indicated that the isolation methods of ovine oviduct epithelial cells were effective.

Quantitative RT-PCR was conducted to determine which type of beta defensin was responsible for the immune reaction in ovine oviduct epithelial cells. SBD-1 mRNA was expressed at a very high level in ovine oviduct epithelial cells, whereas that of SBD-2 was not detectable (Additional file 1: Figure S1).

The results of quantitative RT-PCR demonstrated that treatment with LPS (10–1000 ng/mL) for 24 h caused a concentration-dependent increase in the SBD-1 mRNA expression (Fig. 1b). The SBD-1 mRNA expression began to increase when the cells were treated with 10 ng/mL LPS (2.0-fold to the blank group, *p* <0.01) and increased dramatically at 50 ng/mL LPS (4.9-fold, *p* <0.01). The maximal induction was observed at 100 ng/mL LPS with a 7.8-fold increase in the SBD-1 mRNA expression (*p* <0.01). In the following experiment, ovine oviduct epithelial cells were treated with 100 ng/mL LPS for different times to determine the kinetics of SBD-1 mRNA expression. The expression of SBD-1 mRNA began to rise when the cells were treated for 3 h (2.3-fold to the blank group of 0 h, *p* <0.01) and rose dramatically at 6 h (6.3-fold, *p* <0.01). The maximal induction was observed at 12 h with a 7.7-fold increase in the SBD-1 mRNA expression (*p* <0.01) (Fig. 1c).

### Role of P38 MAPK in LPS-induced SBD-1 mRNA expression

Western blotting was performed to investigate the role of LPS in the activation of MAPKs in ovine oviduct epithelial cells. The results showed that all of the MAPK families were activated after the treatment with 100 ng/mL LPS. The apparent activation of P38 MAPK occurred at 20 min (*p* <0.01), while JNK and ERK were markedly activated at 10 min. Furthermore, phosphorylated P38 MAPK and ERK decreased dramatically at 40 min, while JNK signaling was still activated by LPS (Fig. 1d, e).

**Fig. 2:**
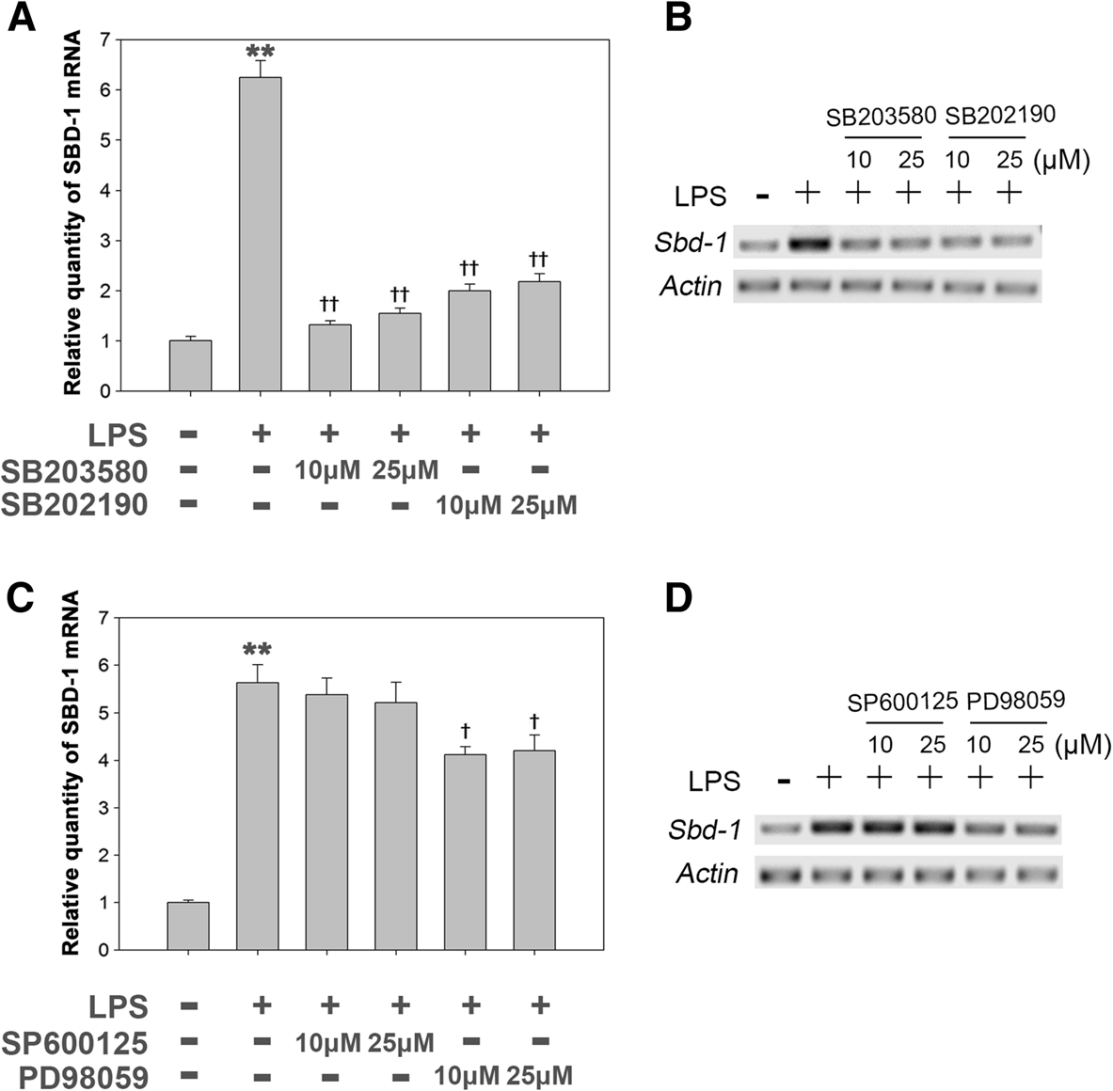
LPS induces SBD-1 expression mainly through P38 MAPK signaling but not through JNK or ERK signaling. **a b** OOECs were cultured with LPS (100 ng/mL), with or without P38 MAPK inhibitors SB203580 and SB202190 for 12 h. Total RNA was prepared and used for examination of SBD-1 mRNA expression by QRT-PCR or RT-PCR with specific primers for SBD-1 and GAPDH. **c d** OOECs were cultured with LPS (100 ng/mL), with or without JNK inhibitor SP600125 and ERK inhibitor PD98059 for 12 h. Total RNA was prepared and used for examination of SBD-1 mRNA expression by QRT-PCR or RT-PCR with specific primers for SBD-1 and GAPDH. All of the experiments were repeated at least three times. *p < 0.05, **p < 0.01 (*t*-test) vs. control. †*p* < 0.05, ††*p* < 0.01 (*t*-test) vs. LPS

To investigate the function of MAPK signaling in the LPS-induced SBD-1 mRNA expression, oviduct epithelial cells were cultured with LPS, with or without MAPK inhibitors. According to the quantitative RT-PCR results, the expression of SBD-1 mRNA induced by LPS was dramatically reduced (2.9–4.7-fold, *p* <0.01) in ovine oviduct epithelial cells pretreated with SB203580 and SB202190, which are selective inhibitors of P38 MAPK signaling, compared to the group treated with LPS alone (Fig. 2a and b). In contrast, LPS-induced SBD-1 expression in ovine oviduct epithelial cells was not affected by the attenuation of JNK signaling (SP600125). Interestingly, the attenuation of ERK signaling (PD98059) slightly decreased (1.3-fold, *p* <0.05) the mRNA levels of SBD-1 induced by LPS (Fig. 2c and d).

### Cellular localization of P38 MAPK

**Fig. 3:**
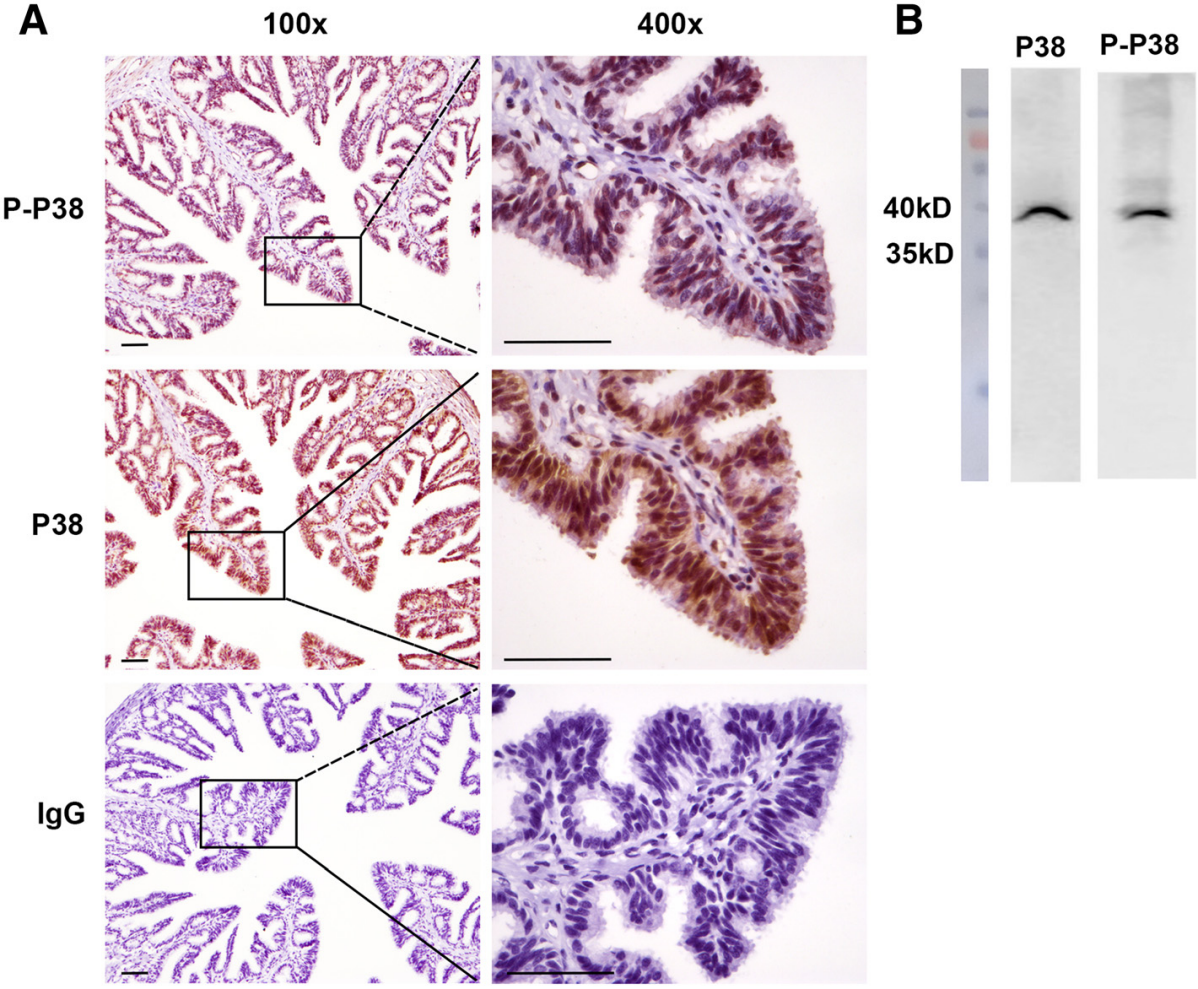
The cellular localization of P38 MAPK in ovine oviducts. **a** Immunohistochemistry was performed to determine the cellular localization of P38 and P-P38 in the ovine oviduct. P38 was detected in the epithelial cells but not the stromal cells in the ovine oviduct. The expression pattern of P-P38 was consistent with that of P38. Ovine oviducts were immunohistochemically stained with rabbit IgG as a negative control. Scale bars: 20 μm. **b** Western blotting experiments were conducted to examine the P38 and P-P38 expression in ovine oviducts in order to detect the specificity of these antibodies

To better understand the role of P38 MAPK during the LPS-induced inflammatory response, the cellular localization of P38 and P-P38 in the ovine oviduct was determined by immunohistochemistry. P38 was present in the epithelial cells but not in stromal cells in the ovine oviduct, as shown in Fig. 3a. Furthermore, the cellular expression of P-P38 colocalized with that of P38. No immunoreactivity was observed in the negative control ovine oviduct, in which the P38 antibody was replaced with a nonspecific rabbit IgG. In addition, as the used antibodies were polyclonal antibodies, western blotting was performed to validate the specificity of these antibodies in OOECs. The results exhibited a clear band at 38 kD (Fig. 3b), which indicated that the protein localized in OOECs by immunohistochemistry analysis were absolutely P38 and P-P38 protein.

### LPS induces the expression of SBD-1 through TLR4

**Fig. 4:**
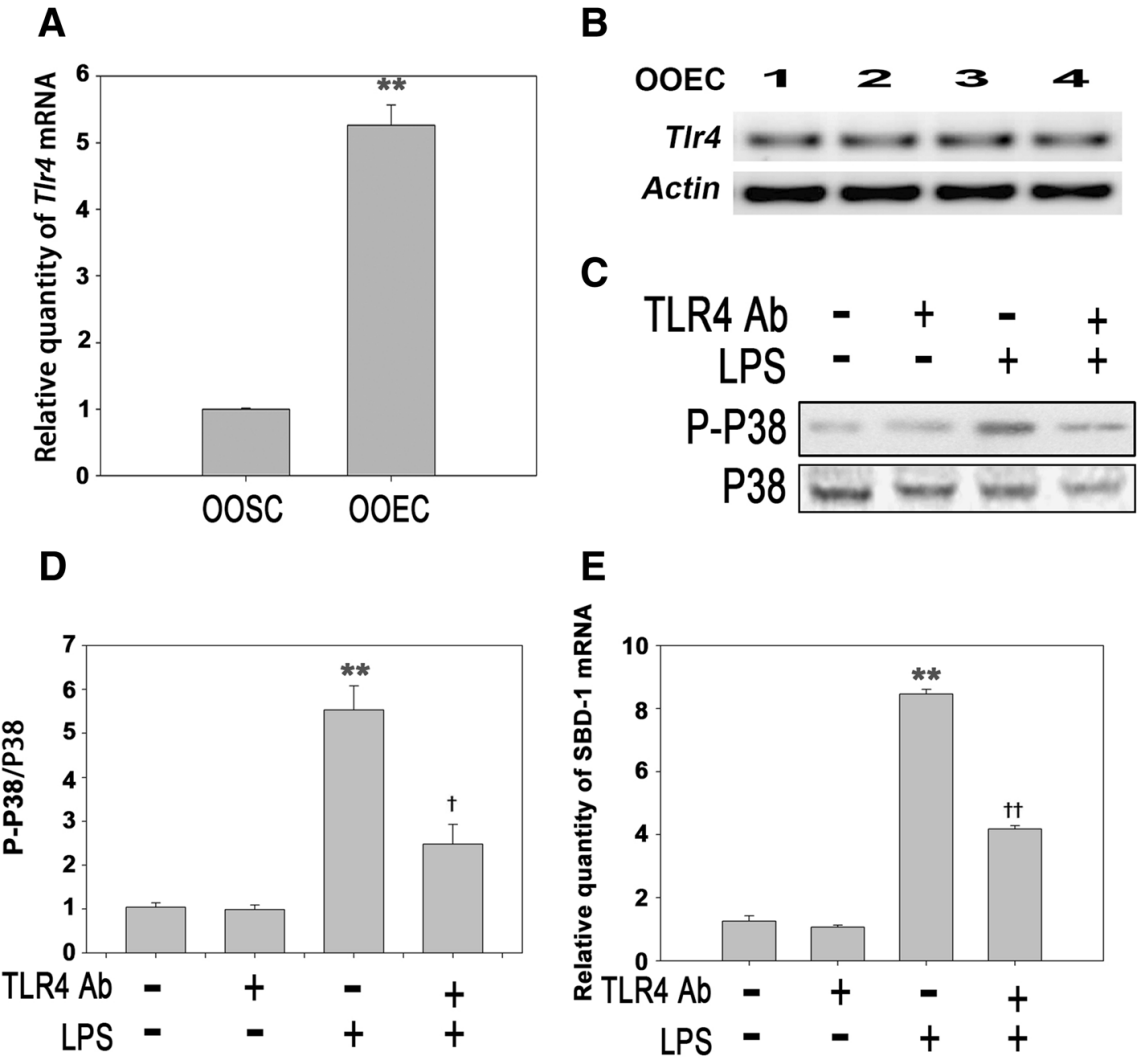
LPS stimulates the expression of SBD-1 through TLR4. **a** *Tlr4* mRNA expression of ovine oviduct stromal cells (OOSCs) and epithelial cells (OOECs) was assessed by qRT-PCR. **b** PCR was conducted to examine the expression of TLR4 in the ovine oviduct epithelial cells harvested at different times. **c** Western blotting was performed to examine the expression levels of P38 MAPK after treatment with LPS (100 ng/mL) or a TLR4 neutralizing antibody for 12 h. **d** The densitometric analysis of the bands on the western blotting showed that LPS could markedly activate P38 MAPK, while the separate addition of the TLR4 neutralizing antibody had no effect. However, treatment with the TLR4 neutralizing antibody could significantly decrease the levels of phosphorylated P38 induced by LPS. **e** QRT-PCR analysis was used to examine the mRNA levels of SBD-1 after treatment with the TLR4 neutralizing antibody for 12 h. Blocking TLR4 activity could inhibit the expression of SBD-1. All of the experiments were repeated at least three times. *p < 0.05, **p < 0.01 (*t*-test) vs. Control. †*p* < 0.05, ††*p* < 0.01 (*t*-test) vs. LPS

TLR4 plays an essential role in initiating the immune system to eliminate invading microbes [19]. The quantitative RT-PCR was performed to detect TLR4 expression in ovine oviduct stromal cells (OOSCs) and epithelial cells (OOECs). The results revealed that TLR4 mRNA was mainly expressed in OOECs compared with that in OOSCs (Fig. 4a, b).

To determine the role of TLR4 in the LPS-stimulated immune response more thoroughly, the cultured ovine oviduct epithelial cells were treated with LPS with or without the TLR4 neutralizing antibody for 12 h. Western blotting was performed to examine the expression levels of P38 MAPK after the treatment with LPS or the TLR4 neutralizing antibody. LPS markedly activated P38 MAPK (*p* <0.01), while the addition of TLR4 neutralizing antibody showed no effect. Interestingly, the treatment with TLR4 neutralizing antibody significantly decreased the levels of phosphorylated P38 induced by LPS (Fig. 4c and d). As previous results demonstrated that LPS induced the expression of SBD-1 through P38 MAPK signaling, the mRNA levels of SBD-1 were examined after the treatment with TLR4 neutralizing antibody. According to hypothesis, blocking TLR4 activity inhibited the expression of SBD-1. The SBD-1 level decreased 2.0-fold when the cells were treated with LPS and anti-TLR4 antibody compared to the SBD-1 expression level in cells treated with LPS only (Fig. 4e).

## Discussion

Microbial pathogen infection triggers the immune response in the ovine oviduct, and epithelial cells are the main cell types that defend against invading pathogens. Defensins play an important role in this immune reaction [25]. LPS, the major cell wall constituent of Gram-negative bacteria, can upregulate the expression of beta-defensins in epithelial cells [26]. However, the regulatory effect of LPS on the expression of beta-defensins in ovine oviduct epithelial cells and the underlying mechanisms remain poorly understood. In this study, we established an in vitro ovine oviduct epithelial cell culturing system and treated the cells with LPS to mimic microbial infection to further understand the effect of LPS on SBD-1 expression and the possible signaling pathway involved.

So far, only beta defensin-1 (BD-1) and beta defensin-2 (BD-2) have been identified in sheep [27]. To determine which type of beta-defensin was responsible for the immune response of ovine oviduct epithelial cells to microbial infection, quantitative RT-PCR was conducted to identify the expression pattern of SBD-1 and SBD-2. Only SBD-1 was found to be expressed at an abundant level in ovine oviduct epithelial cells in a concentration- and time-dependent manner, whereas SBD-2 was not detectable after the treatment with LPS.

According to the classical LPS-mediated cell signaling pathway, LPS could bind to LPS-binding proteins (LBP) in plasma, and the LPS-LBP complex interacted with CD14 to form a ternary complex, named LPS-LBP-CD14 to transfer LPS to the TLR4 accessory protein MD2 complex. This transfer led to the activation of TLR4. LPS-activated TRL4 activated the NF-kB signaling pathway and all the three MAPK pathways, including ERK, JNK/SAPK and p38 [28–30]. The MAPK family mediated the LPS-induced expression of beta defensin. The MEK1/2-ERK signaling pathway was required for the upregulation of defensin 2 in human middle-ear epithelial cells [31]. P38 MAPK and JNK were involved in the regulation of beta defensin 2 in A549 cells [32]. In the present study, 100 ng/mL LPS stimulated the phosphorylation of ERK, P38 MAPK and JNK, and the activation of these kinases was apparent at 10 min. Furthermore, the pretreatment with a specific inhibitor of P38 MAPK drastically decreased the SBD-1 expression, induced by LPS. The immunohistochemistry results further indicated the existence of P38 MAPK in ovine oviduct epithelial cells. It was also found that ERK might slightly regulate SBD-1, while JNK signaling showed no effect. These results suggested that the upregulation of SBD-1 by LPS may be a programmed process which required the activation of P38 MAPK.

In this study, we also demonstrated that TLR4 was highly expressed in ovine oviduct epithelial cells, and the attenuation of TLR4 activity markedly inhibited the activation of P38 MAPK and the downstream upregulation of SBD-1.

Oviduct pathogen infection is thought to be the leading cause of tubal infertility [33]. The innate immune system of the female reproductive tract, such as defensin and pattern recognition toll-like receptors, plays a pivotal role in pathogen resistance [34]. It was demonstrated that chicken oviduct epithelial cells express most of the known defensin genes, and these defensins effectively function against pathogen infection [35, 36]. Also, defensins were localized in ovine oviduct epithelium in response to bacterial infection, such as *Chlamydia/Chlamydophila* [37, 38]. To investigate the interplay between bacterial infection and defensin stimulation, LPS, the major constitute of gram-negative bacteria, was added into OOECs culturing system in order to mimic a bacteria induced immune response. So far, LPS-TLR4-P38 MAPK pathway was firstly founded by us to control inner defensin immunity in ovine oviduct. However, a specific tubal disease model is needed to establish for a further research, and relative medicine targeting the LPS-TLR4-P38 MAPK pathway might be used in clinic.

## Conclusions

**Fig. 5:**
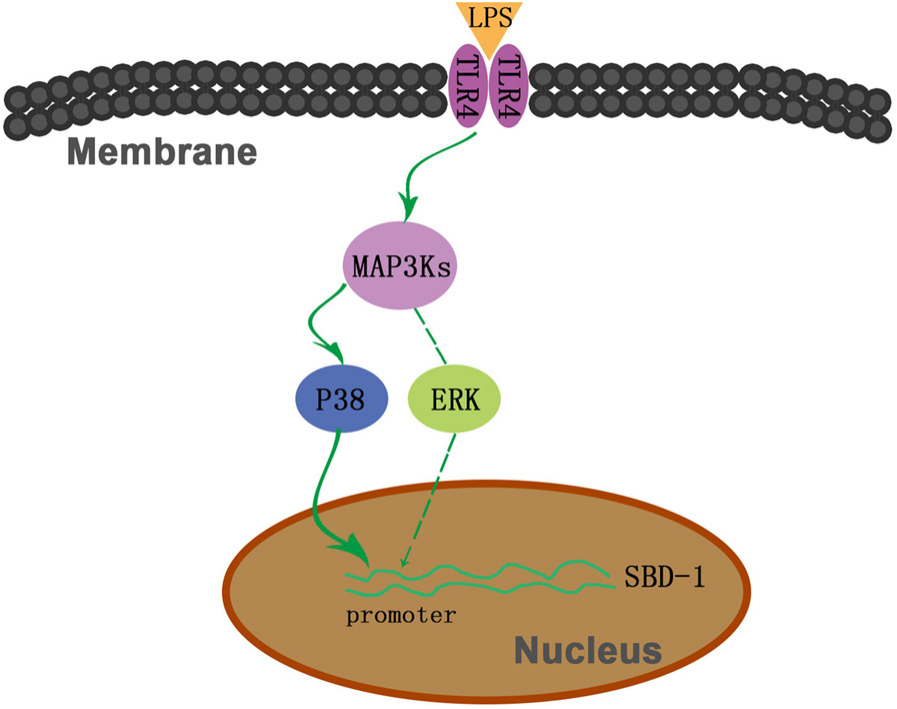
Schematic diagram of LPS regulating SBD-1 expression. LPS binds to TLR4 receptor, activates P38 MAPK signaling pathway, and subsequently stimulates SBD-1 expression in ovine oviduct epithelial cells

In conclusion, findings of the present study demonstrate that LPS induces SBD-1 mRNA expression in ovine oviduct epithelial cells via activation of p38 MAPK. TLR4 receptor may involve in LPS-induced p38 MAPK activation (Fig. 5). We believe that these observations may have broad implications for the understanding of the pathogenesis of oviduct inflammation, such as salpingitis, and the reaction of the host immune system to microbial invasion.

## Abbreviations

BSA, bovine serum albumin; DMEM/F12, Dulbecco’s modified eagle medium: nutrient mixture F-12; ERK, extracellular signal-regulated kinase; HBSS, Hanks balanced salt solution; JNK, c-Jun N-terminal kinase; LBP, LPS-binding proteins; LPS, lipopolysaccharide; MAPKs, mitogen-activated protein kinases; OOECs, ovine oviduct epithelial cells; OOSCs, ovine oviduct stromal cells; PBS, phosphate-buffered saline; qRT-PCR, quantitative RT-PCR; SBD-1, sheep β-defensin-1; TLR4, Toll-like receptor 4

## Additional file

Additional file 1: Figure S1. The mRNA levels of SBD-1 and SBD-2 in ovine oviduct epithelial cells. The quantitative RT-PCR results showed that SBD-1 mRNA was expressed at a very high level in ovine oviduct epithelial cells, while SBD-2 mRNA was not detectable. All of the experiments were repeated at least three times. **p* < 0.05, ***p* < 0.01 (*t*-test) vs. SBD-1. (TIF 128 kb)
