# Supplementary figures and images for: Lipopolysaccharide induces SBD-1 expression via the P38 MAPK signaling pathway in ovine oviduct epithelial cells
Source: Lipids Health Dis. 2016 Aug 11;15:127. doi: 10.1186/s12944-016-0294-4 (PMC4981948; doi:10.1186/s12944-016-0294-4)

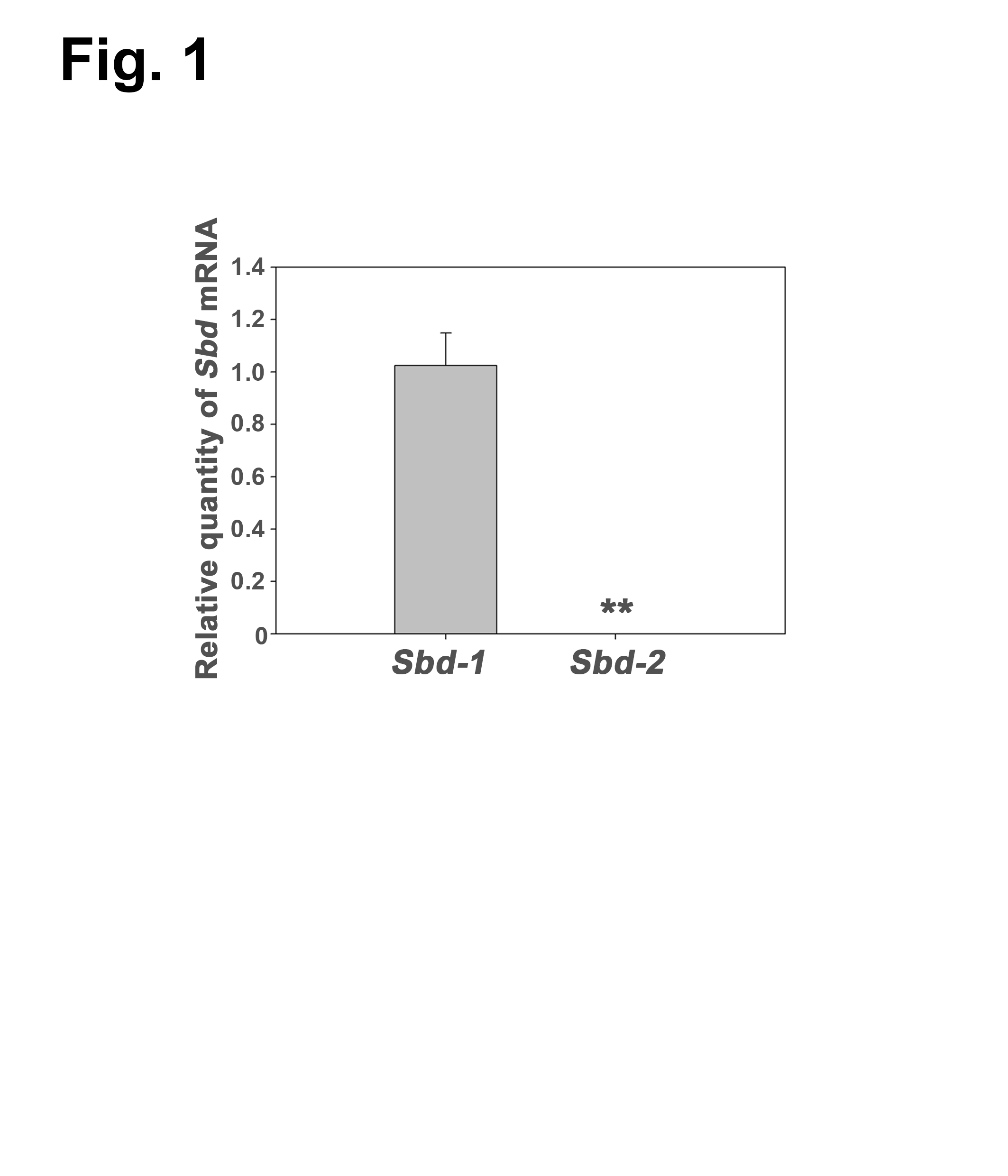

Supplement: Additional file 1: Figure S1. — The mRNA levels of SBD-1 and SBD-2 in ovine oviduct epithelial cells. The quantitative RT-PCR results showed that SBD-1 mRNA was expressed at a very high level in ovine oviduct epithelial cells, while SBD-2 mRNA was not detectable. All of the experiments were repeated at least three times. *p < 0.05, **p < 0.01 (t-test) vs. SBD-1. (TIF 128 kb) [file 12944_2016_294_MOESM1_ESM.tif]
